# Rivaroxaban Versus Warfarin for the Treatment of Cerebral Venous Thrombosis (RWCVT): A Randomized Controlled Trial in Resource-Limited Setting

**DOI:** 10.1155/srat/8893742

**Published:** 2025-05-05

**Authors:** Ahmad Alkhawam, Lina Okar, Ibrahem Hanafi, Peyton Murin, Ali Ibrahim, Juman Isstaif, Eman Khashaneh, Rami Z. Morsi, Tareq Kass-Hout

**Affiliations:** ^1^Faculty of Medicine, University of Damascus, Damascus, Syria; ^2^Department of Neurology, SSM Health/Saint Louis University, St. Louis, Missouri, USA; ^3^Department of Neurology, Damascus Hospital, Damascus, Syria; ^4^Department of Neurology, University of Chicago, Chicago, Illinois, USA

## Abstract

**Background:** Cerebral venous thrombosis (CVT) is a rare but potentially debilitating form of stroke. Current management guidelines recommend a course of low molecular weight heparin (LMWH) followed by an oral vitamin K antagonist. However, there is an emerging body of evidence to support the use of direct oral anticoagulant (DOAC) medications. Here, we assess the efficacy of rivaroxaban compared to the standard of care in a resource-limited setting.

**Methods:** The study was designed as a Phase III, prospective, parallel, open-label, randomized controlled trial conducted in three sites in Syria. Seventy-one participants met inclusion criteria and were randomized 1:1 to receive either rivaroxaban or warfarin following initial bridging with LMWH for 3.5–12 days. The primary outcome was functional improvement determined by the Barthel Index. Secondary outcomes were adverse events during follow-up, including CVT recurrence, thrombotic events, intracranial pressure (ICP) requiring shunt placement, extra and intracranial bleeding, neurological deficit, and all-cause mortality.

**Results:** Barthel Index scores did not differ between the study cohorts at 1-, 2-, 3-, 4-, 5-, or 6-month follow-up. Secondary analysis yielded no difference in rates of adverse effects or return of CVT. Two patients in the warfarin group developed major extracranial bleeds (uterine bleeding); however, there were no other extracranial or intracranial bleeds or thrombotic events reported. Rates of all-cause mortality and all assessed adverse effects were similar between the groups.

**Conclusion:** We offer a prospective, parallel randomized controlled trial that suggests rivaroxaban may have comparable safety and efficacy when compared to warfarin for the treatment of CVT. Importantly, we offer the first randomized control trial of oral anticoagulants for the treatment of CVT in a resource-limited setting, providing support for the evolving literature and suggesting the safety and efficacy of oral anticoagulants in the management of CVT.

**Trial Registration:** ClinicalTrials.gov identifier: NCT04569279

## 1. Introduction

Cerebral venous thrombosis (CVT) is a rare subtype of stroke, affecting cerebral veins or dural sinuses. While CVT comprises only 0.5%–1% of all strokes, CVT can be debilitating, resulting in lifelong disability in up to 15% of patients [1]. Pathophysiologically, CVT manifests as diminished venous blood drainage and cerebrospinal fluid (CSF) absorption, causing elevation in the intracranial pressure (ICP) and/or cerebral parenchymal lesions or dysfunction [2]. Clinical presentation includes headache, seizure, papilloedema, focal neurological deficits, and/or altered mental status [3].

CVT risk factors are similar to venous thromboembolic events (VTEs), including irreversible factors like inherited thrombophilia and transient factors such as oral contraceptives, puerperium, malignancies, and infectious diseases [2]. Diagnosis of CVT poses challenges due to its common risk factors, diverse clinical presentations, and sometimes equivocal neuroimaging; however, enhanced brain magnetic resonance imaging (MRI) with susceptibility-weighted imaging (SWI) sequence remains the optimal diagnostic modality [4].

Prompt initiation of anticoagulation therapy with unfractionated heparin (UFH) or low molecular weight heparin (LMWH), followed by a variable duration of oral anticoagulation, traditionally warfarin, represents the standard of care in CVT [4]. However, rivaroxaban and other direct oral anticoagulants (DOACs) have emerged as a promising alternative to warfarin. Benefits of DOACs are selective inhibition of factor X and reduced need for laboratory monitoring or dose adjustments [5].

Previously, the SECRET trial, the RESPECT CVT trial, and EINSTEIN Jr-CVT have suggested the newest anticoagulants may be a safe and efficacious alternative to warfarin in the management of CVT [6–8]. However, these previous trials have been conducted in a resource-rich setting in the Western world. Previous literature has suggested that rates of CVT may be higher in other geographic regions, suggesting a need for safe and efficacious treatment within this region [9]. To this end, we sought to establish the safety and efficacy of rivaroxaban for the management of CVT within a resource-limited setting.

## 2. Methods

### 2.1. Data Sharing Statement

Deidentified participant data will be available once planned subanalyses are complete, on request following an approved proposal and with a signed data access agreement. Requests should be emailed to the corresponding author.

### 2.2. Compliance Statement

IRB approval was obtained (IRB approval number: 38). The trial reporting was structured in keeping with the CONSORT 2010 guidelines.

### 2.3. Patient Recruitment

RWCVT was conducted as an open-label, multicenter, parallel, RCT. Seventy-one patients were recruited from three tertiary hospitals in Damascus (Al-Mouassat, Al-Assad, and Al Mujtahid hospitals) between September 2017 and September 2019. Written informed consent was obtained from participants, or, when participants were unable to directly provide consent, from a legally authorized representative. Once enrolled, the patients were followed for 6 months, with the last follow-up visit completed in March 2020. Patient demographics and detailed clinical information, including CVT-provoking factors, affected vessel, presenting symptoms, and other medications received, were collected from charts. Enrollment was done based on a convenience sample within the IRB-approved time window.

Inclusion criteria were as follows: age > 14 years or older, weight > 50 kg, and a recent (< 7days) diagnosis of symptomatic CVT, confirmed by imaging (computed tomography venography or MRI venography).

Exclusion criteria were as follows: contraindications to anticoagulation use, severely impaired renal function (creatinine clearance (CrCl) < 30 mL/min using the Cockcroft–Gault equation), pregnancy or lactation at randomization, and concomitant mandatory use of another anticoagulant/platelet.

### 2.4. Interventions and Randomization

All patients initially received a weight-adjusted dose of LMWH ranging from 3.5 to 12 days until able to take oral medications. Confirmation of acute/subacute CVT was done using imaging 24–48 h from admission, after which patients were randomized at 1:1 to receive either rivaroxaban or warfarin. Randomization was done using a random number generator. Rivaroxaban dose of 20 mg daily (or 15 mg if CrCl was 30–50 mL/min) and warfarin was titrated to an international normalized ratio (INR) of 2–3. Patients noted to have elevated ICP received acetazolamide at a dose of 500 mg twice a day for at least 2 weeks, followed by gradual tapering, regardless of the treatment group.

**Figure 1:**
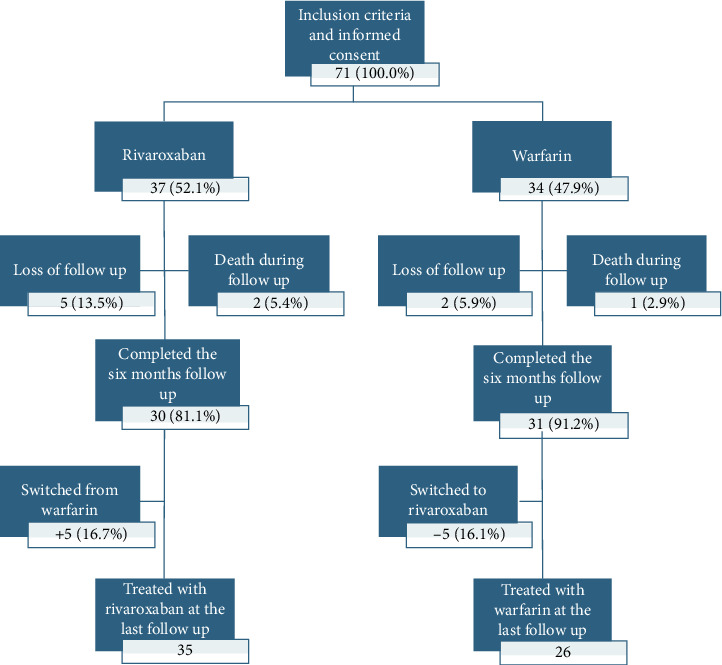
Flow chart of patient randomization and follow-up.

The primary outcome was functional improvement determined by the Barthel Index. Secondary outcomes were papilledema, resolution of ICP increase, and adverse outcomes. This was reported systematically using a research form during follow-up; this included CVT recurrence, thrombotic events, ICP requiring shunt placement, extra and intracranial bleeding, neurological deficit, and all-cause mortality. Patients were assessed using the sinus venous thrombosis severity scale (SVTSS) at treatment initiation and the Barthel Index at initiation, discharge, and monthly during follow-up [10] (Supporting Information 2: Appendix 1). Two or more investigators evaluated each patient to affirm objective and accurate assessment of their data and outcome measures and to reduce the impact of possible inter-rater variability. Assessment of papilledema was done by a neurologist and ophthalmologist at onset, with a follow-up assessment done by an ophthalmologist. While it would have been ideal to obtain repeat imaging, given the sociopolitical climate in the study country at the time, such follow-up was not able to be reliably obtained (Figure 1).

### 2.5. Statistical Analysis

All statistical analysis was done using Prism Version 9.5.1 (GraphPad Software, San Diego, CA). Patient age and length of LMWH were reported as mean and interquartile range (IQR). SVTSS scores and Barthel Index scores were reported as a mean and standard deviation (SD). Patient gender, provoking factors, baseline symptoms, affected vessel, presenting symptom, medications received, and adverse effects were reported as percentages. The parametric *p* value was calculated using chi-square for categorical variables. The nonparametric *p* value was calculated by the Mann–Whitney *U* test for continuous variables.

Comparisons between groups for the Barthel Index were assessed using a Kaplan–Meier survival analysis. One patient had severe psychomotor retardation prior to presentation and, as such, was considered an outlier and was excluded from Barthel Index analysis. Analysis of papilledema was done using the Kaplan–Meier survival analysis, with a favorable outcome defined as the resolution of papilledema. Major complications of treatment were defined consistently with the criteria of the International Society of Thrombosis and Hemostasis (ISTH).

For all statistical analyses, patients were analyzed using an intention-to-treat approach with an alpha value of 0.05 as the threshold for statistical significance.

## 3. Results

### 3.1. Patient Characteristics and Clinical Presentation

A total of 71 patients were recruited with an average age of 33 years and a sex distribution of 31% male, 69% female. Patients within the rivaroxaban group were on average older (35.5 vs. 30.0 years; *p* = 0.0329); however, no other demographic differences were noted. In both the R and W group, the majority of CVTs were provoked (56.8% and 61.8%); however, there were no statistically significant differences in the percentage of provoked or provoking factors between the groups (Table 1). The superior sagittal sinus was more commonly affected in the rivaroxaban group (75.7% vs. 38.2%; *p* = 0.0014); however, there were no differences in any of the other affected vessels. Severity at initiation of treatment, presenting symptoms, and use of acetazolamide or corticosteroids did not differ between the groups. Of note, corticosteroids were used for another indication and not for managing CVST. The average duration of LMWH was 9.0 days in the warfarin group compared to 6.0 days in the rivaroxaban group (*p* = 0.0030). While no patients in the study had a medical history of a clotting disorder, two patients (one in the R group and one in the W group) had a family history of a clotting disorder.

### 3.2. Follow-Up Evaluation

**Figure 2:**
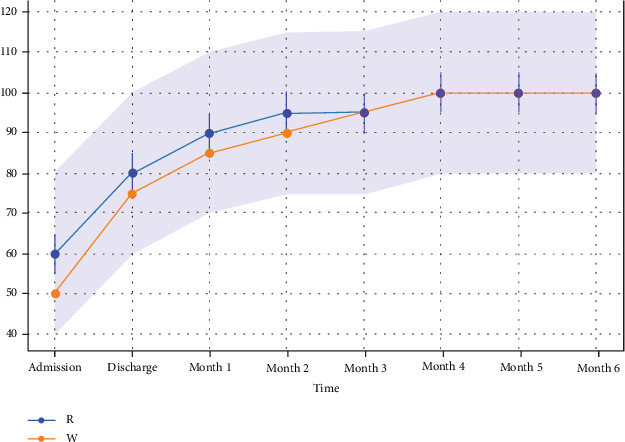
Barthel Index scores versus time. The orange and blue lines represent the mean score in the warfarin and rivaroxaban arms, respectively, for each follow-up assessment time point. The light blue area is the range of mean score ±20% of the maximum measured score in the warfarin arm. The error bars for the blue line represent the 95% confidence interval in the rivaroxaban arm at each assessment. Importantly, disability by the Barthel Index displayed no statistically significant differences between the study groups.

**Figure 3:**
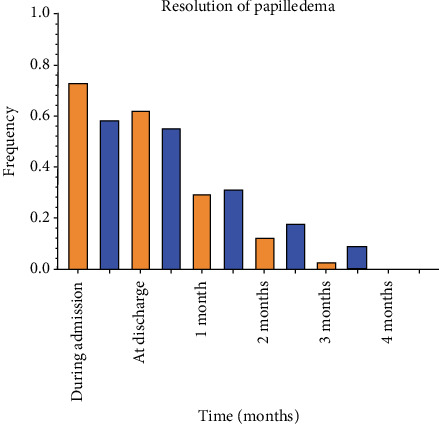
Resolution of papilledema.

No statistically significant differences were observed in Barthel Index scores at initiation, discharge, or follow-up at 1–6 months (Figure 2). The Kaplan–Meier survival analysis, followed by Cox regression, was performed to assess papilledema (Figure 3).

The mean time to resolution of papilledema was 2.0 (1.4–2.5) and 1.4 (1.0–1.9) months for the warfarin and rivaroxaban arms, respectively. Cox regression revealed a hazard ratio of 1.41 (0.77–2.59), which did not reach statistical significance (*χ*^2^ = 1.24, *p* = 0.27). (Figure 3).

### 3.3. Adverse Effects

The most common adverse effect noted was the need for a ventriculoperitoneal shunt, occurring in 4.2% of the patients. At the end of the follow-up period, seven patients did not reach total recovery, three had residual optic nerve deficits, two developed chronic migrainous headache, and one had oculomotor nerve deficits. No statistically significant differences were observed in rates of adverse effects or repeat CVT between the groups (Table 1). It is pertinent to note that, in the warfarin group, the oral anticoagulant dose was modified 125 times in 26 patients (78.7%) during the follow-up period. There were two major extracranial bleeds in the warfarin group, both involving uterine bleeding. No other major or minor extracranial or intracranial hemorrhages, or new thrombotic events, occurred during the follow-up period. Three patients died (two due to malignancies and one from the initial severe CVT), and seven patients were lost to follow-up (five in the rivaroxaban group and two in the warfarin group) (Table 1).

## 4. Discussion

In RWCVT, we found no difference in functional disability as assessed by the Barthel Index in patients treated with rivaroxaban when compared to warfarin. Furthermore, we observed a comparable safety profile, with no statistically significant differences in rates of adverse effects or recurrence of CVT. The authors did not note any statistically significant differences in time to resolution of papilledema between the study groups. On the whole, due to the need for bridging, warfarin patients had a longer length of stay and required frequent dose adjustments, suggesting a possible logistical benefit with the use of DOACs.

Similar to the evolving literature on the use of DOAC in CVT, our study emphasizes the efficacy and safety of rivaroxaban when compared to warfarin. In the SECRET trial, a Phase II RCT that compared rivaroxaban to warfarin in patients with CVST in 53 patients, the authors reported overall low rates of bleeding and recurrent venous thrombosis [6]. Of note, one major and two nonmajor bleeding events in the SECRET trial were in the rivaroxaban arm. In the RESPECT-CVT trial, another RCT, 120 patients were randomized to either dabigatran or warfarin after 5–15 days of parenteral heparin treatment; they reported no recurrence of venous thrombosis and only three major bleeding events, one in the dabigatran group and two in the warfarin group, with one additional nonmajor bleeding event in the warfarin arm. The outcome suggested that both warfarin and dabigatran are effective and safe in CVT [7]. EINSTEIN Jr-CVT is an RCT that compared rivaroxaban to warfarin in pediatrics, included 500 patients, and concluded that treatment with rivaroxaban has a similar lower risk of CVT recurrence without increased bleeding risk; only 3% of 329 children on rivaroxaban had nonmajor bleeding compared to 2% of 162 children in the warfarin group (two major and one nonmajor) [11]. In RWCVT, two major, yet extracranial bleeds, were reported in the warfarin group with no bleeding events in the rivaroxaban group. Additionally, similar to SECRET and EINSTEIN Jr-CVT, our patients received a 20 mg dose of rivaroxaban vs. 15 mg if they had low CrCl without any safety concerns regarding this starting dose [6, 11]. ACTION-CVT is an international multicentric retrospective study that included 845 patients and concluded that DOAC is associated with similar clinical and radiographic outcomes with favorable safety profiles. Rivaroxaban and dabigatran showed similar rates of major bleeding compared to warfarin but lower risk of intracranial hemorrhage [12].

While previous studies have looked primarily at functional recovery using the Modified Rankin Scale (mRS), our study used an alternative, but established measure of functional disability, the Barthel Index [13]. In RWCVT, we chose to use the BI as it was specifically designed as a detailed assessment of activities of daily living, enabling us to capture changes in functional status over monthly follow-up [14]. Importantly, the Barthel Index has been shown to correlate with mRS, the more often used measure in similar studies [15].

When compared to the previous literature, our study is unique in that it is the first to explore this problem in a resource-limited setting. While previous studies have consisted only of sites in resource-rich nations, our study was conducted in Syria during the conflict. We find this important as there is evidence to suggest the burden of disability from stroke and other vascular causes is disproportionately high in low- and middle-income nations [16]. Furthermore, while previous studies have used imaging to follow patients, routine imaging follow-up is not always available outside of these settings [17]. In these cases, guidance is limited.

Our study is the first to attempt to address this complex clinical problem within a resource-limited setting. We find this particularly pertinent, as the literature would suggest that rates of CVT may be higher within the geographic region of the study, suggesting a need for safe and efficacious treatment [9].

Additional strengths of the study include the prospective design, the robust sample size when considering the overall low incidence of CVT, the inclusion of multiple centers, and the frequency of follow-up assessment. Limitations include the open-label design with the inability to blind providers to treatment due to patient safety, a possible source of bias, and the limited statistical power (*n* = 71). Barthel Index is limited by its U-shaped distribution in diverse disability cohorts, but this likely did not affect our findings due to the predominance of mild to moderate disability in our patients. The limited analysis on medical comorbidities, limited follow-up (only 88.7% completed all follow-up visits), and the lack of radiographic follow-up tests were the limitations of the study.

## 5. Conclusion

CVT represents a neurological emergency with high morbidity and mortality. The standard of care is currently heparin followed by vitamin K antagonist; however, there is an emerging body of literature to suggest the use of DOACs as a therapeutic alternative. A limitation to broader clinical adoption remains the lack of prospective clinical trials. To this end, we share a multicenter, open-label, Phase III, parallel RCT, showing no statistically significant differences in disability nor adverse effects between rivaroxaban and warfarin for the management of CVT. Importantly, our study is the first to investigate this clinical question outside of a resource-rich nation, providing guidance for practitioners in low- and middle-income countries.

## Figures and Tables

**Table 1:** Patient characteristics.

	**All patients**	**Rivaroxaban group**	**Warfarin group**	**p** ** value**
Number of patients	71	37	34	
Gender				
Male (%)	31.0%	24.3%	38.2%	0.2054
Female (%)	69.0%	75.7%	61.8%	0.2054
Age (median years, 95% CI)	33.0 (27.0–36.0)	35.5 (27.0–39.0)	20.0 (21.0–36.0)	0.0329⁣^∗^
Venous sinus thrombosis				
Provoked (%)	59.1%	56.8%	61.8%	0.6680
Unprovoked (%)	40.9%	43.2%	41.2%	0.8602
Provoking factors				
Oral contraceptives (%)	43.9%	32.4%	26.5%	0.5824
Peripartum state (%)	21.4%	8.1%	11.8%	0.6056
Cranial infection (%)	9.7%	5.4%	5.9%	0.9306
Baseline symptoms				
SVTSS (mean, 95% CI)	9.4 (8.3–10.6)	9.5 (8.0–11.0)	9.4 (7.5–11.2)	N/A
Barthel Index (mean, 95 CI)	59.5 (51.3–67.7)	55.0 (44.3–65.8)	64.3 (51.4–77.0)	N/A
Elevated ICP (%)	66.2%	73.0%	58.8%	0.2080
ICH (%)	32.4%	35.1%	32.4%	0.8045
Affected vessel				
Superior sagittal sinus (%)	64.7%	75.7%	38.2%	0.0014⁣^∗^
Right transverse sinus (%)	45%	46.0%	32.4%	0.2417
Left transverse sinus (%)	38.0%	35.1%	38.2%	0.7865
Isolated cortical vein (%)	5.6%	10.8%	14.7%	0.6222
Presenting symptom				
Headache (%)	100.0%	100.0%	100.0%	N/A
Focal neurological signs (%)	67.6%	64.9%	70.6%	0.6067
Seizures (%)	53.5%	51.4%	55.9%	0.7022
Status epilepticus (%)	54.9%	65.0%	42.9%	0.0792
Altered consciousness (%)	5.6%	4.8%	5.0%	0.9306
Coma (%)	8.5%	8.1%	8.8%	0.9138
Medications received				
Acetazolamide (%)	63.4%	62.2%	64.7%	0.8241
Corticosteroids (%)	16.9%	13.5%	20.6%	0.4268
LMWH days (median days, 95% CI)	7.0 (6.0–9.0)	6.0 (4.0–7.0)	9.0 (7.0–10.0)	0.0030⁣^∗^
Adverse effects				
CVT recurrence (%)	0.0%	0.0%	0.0%	N/A
VP shunt (%)	4.2%	2.7%	5.9%	0.5058
Extracranial bleed (%)	2.8%	0.0%	5.9%	0.1345
Intracranial bleed (%)	0.0%	0.0%	0.0%	N/A
New thrombotic event (%)	0.0%	0.0%	0.0%	N/A
Migraine type headache (%)	2.8%	2.7%	2.9%	N/A
Optic nerve deficit (%)	4.2%	2.7%	5.9%	N/A
Oculomotor nerve deficit (%)	1.4%	0.0%	2.9%	N/A
Psychomotor retardation (%)	1.4%	0.0%	2.9%	N/A
All-cause mortality (%)	4.2%	5.4%	2.9%	N/A
Loss of follow-up	9.9%	13.5%	5.9%	0.2813

*Note:* The parametric *p* value is calculated by the chi-square test for categorical variables. The nonparametric *p* value is calculated by the Mann–Whitney *U* test for continuous values.

⁣^∗^*p* < 0.05.

## Data Availability

The data that support the findings of this study are available from the corresponding author upon reasonable request.

## Nomenclature

RWCVT: rivaroxaban versus warfarin in the management of cerebral venous thrombosis
CVT: cerebral venous thrombosis
DOACs: direct oral anticoagulants
SVTSS: sinus venous thrombosis severity scale
mRS: Modified Rankin Scale
GCS: Glasgow Coma Scale
ACTION-CVT: anticoagulation treatment in cerebral venous thrombosis
SECRET-CVT: safety and efficacy of rivaroxaban in cerebral venous and dural sinus thrombosis
EINSTEIN Jr: evaluation of novel oral anticoagulant rivaroxaban in pediatric patients with venous thromboembolism

## Supporting Information

Supporting Information 1 Consort checklist 2010.
