# Supplementary material for: Rivaroxaban Versus Warfarin for the Treatment of Cerebral Venous Thrombosis (RWCVT): A Randomized Controlled Trial in Resource-Limited Setting
Source: Stroke Res Treat. 2025 May 5;2025:8893742. doi: 10.1155/srat/8893742 (PMC12081145; doi:10.1155/srat/8893742)
Supplement: Supporting Information 2 — Appendix 1. [file 8893742.f2.docx]

**Appendix 1**

*Further definition of the SVTSS components:*

**Headache** severity was estimated using the Functional Pain Scale (FPS) (20) based on the pain tolerability and interference with activities; Slight (2), Moderate (4 – 6), Severe (8 – 10).

**Focal signs** were classified as transient if they lasted for less than 24 hours. The paresis was considered mild when it could only be perceived by comparing with the other side, moderate when it was obvious, but the limb could still move against gravity, severe when the limb could not move against gravity, or it qualifies for complete plegia.

**Cranial nerve defects** were considered as focal signs which were defined as mild for minimal and barely detected defects, moderate for obvious defects, and severe if they led to functional disability such as visual loss. Abducens nerve palsy was always considered as “mild paresis” regardless of functional disability as it is usually a pseudo-localization sign. Patients were scored upon the most severe focal sign when several lesions were evident.

**Seizures** were considered if at least one clinical convulsive seizure was evident, which was considered as an indication of long-term use of anticonvulsant drugs for a minimum of the whole follow-up period. Obvious other cause(s) of seizures not related to a new complication of the disease or anticoagulation treatment (e.g. poor compliance with anticonvulsant drugs) would not affect the severity scale score.

**Consciousness**: According to Dorland's Illustrated Medical Dictionary 32nd edition (21) the investigators defined the following degrees: Confusion: disturbed orientation in regard to time, place, or person; Psychosis: a mental disorder characterized by gross impairment in reality testing as evidenced by delusions, hallucinations, markedly incoherent speech, or disorganized and agitated behavior; Somnolence: drowsiness or sleepiness, particularly in excess; Stupor: a lowered level of consciousness manifested by the subject's responding only to vigorous stimulation; Coma: a state of unconsciousness from which the patient cannot be aroused, even by powerful stimulation; Death.
